# Long Telomeric Repeat-Containing RNA (TERRA): Biological Functions and Challenges in Vascular Aging and Disease

**DOI:** 10.3390/biomedicines11123211

**Published:** 2023-12-03

**Authors:** Paola Canale, Jonica Campolo, Andrea Borghini, Maria Grazia Andreassi

**Affiliations:** 1 CNR Institute of Clinical Physiology, 56124 Pisa, Italy; paola.canale@santannapisa.it (P.C.); andrea.borghini@cnr.it (A.B.); 2 Health Science Interdisciplinary Center, Sant’Anna School of Advanced Studies, 56124 Pisa, Italy; 3 CNR Institute of Clinical Physiology, ASST Grande Ospedale Metropolitano Niguarda, 20142 Milano, Italy; jonica.campolo@cnr.it

**Keywords:** TERRA, telomere, clonal hematopoiesis of indeterminate potential vascular aging, cardiovascular disease

## Abstract

Telomere dysfunction is implicated in vascular aging and shorter leucocyte telomeres are associated with an increased risk of atherosclerosis, myocardial infarction, and heart failure. Another pathophysiological mechanism that explains the causal relationship between telomere shortening and atherosclerosis development focuses on the clonal hematopoiesis of indeterminate potential (CHIP), which represents a new and independent risk factor in atherosclerotic cardiovascular diseases. Since telomere attrition has a central role in driving vascular senescence, understanding telomere biology is essential to modulate the deleterious consequences of vascular aging and its cardiovascular disease-related manifestations. Emerging evidence indicates that a class of long noncoding RNAs transcribed at telomeres, known as TERRA for “TElomeric Repeat-containing RNA”, actively participates in the mechanisms regulating telomere maintenance and chromosome end protection. However, the multiple biological functions of TERRA remain to be largely elucidated. In particular, the role of TERRA in vascular biology is surprisingly unknown. In this review, we discuss the current knowledge of TERRA and its roles in telomere biology. Additionally, we outline the pieces of evidence that exist regarding the relationship between TERRA dysregulation and disease. Finally, we speculate on how a comprehensive understanding of TERRA transcription in the cardiovascular system may provide valuable insights into telomere-associated vascular aging, offering great potential for new therapeutic approaches.

## 1. Introduction

Vascular aging is recognized as one of the hallmarks of atherosclerosis and its acute thrombotic events, such as myocardial infarction, stroke, and ischemic heart failure [1,2]. Indeed, chronological aging plays a significant role in promoting structural and functional changes to the vasculature, and it is one of the major risk factors for atherosclerotic disease. However, lifestyle, environment, and extrinsic stimuli can also cause a premature loss of structure and function in the vascular system with the accumulation of subclinical early vascular aging [1,2]. Therefore, delaying or reversing the deleterious consequences of vascular aging may represent a promising strategy to prevent the risk of developing disease, but it is crucial to better understand the cellular and molecular mechanisms of action [1,2,3]. Over the past decades, growing evidence has shown that telomere length plays a key role in cardiovascular disease by driving cellular senescence and that it elicits the senescence-associated secretory phenotype (SASP) in vascular cells [4]. Cells with SASP produce different autocrine and paracrine factors (growth factors, cytokines, adhesion molecules, and proteases) that promote chronic inflammatory and increased oxidative stress, leading to endothelial dysfunction, cellular proliferation, migration, and ultimately cardiovascular pathologies, such as atherosclerosis, cardiac remodeling, and heart failure. Recently, the discovery of a long noncoding RNA, called TERRA (telomeric repeat-containing RNA), transcribed at chromosome ends [5], has provided new insights into the complexity of telomere homeostasis and telomere functions. Understanding the biology and the deleterious consequences of telomere dysfunction in relation to vascular and cardiac function may have a profound impact on early prevention, risk stratification, and potential new therapeutic intervention.

In this review, we will briefly discuss the current knowledge of TERRA and its roles in telomere biology. Additionally, we will outline the pieces of evidence that exist regarding the relationship between TERRA and disease. Finally, we will speculate on how a better understanding of TERRA transcription in vascular aging may provide great potential in terms of therapeutic strategies.

## 2. Telomere Structure and Function

**Figure 1:**
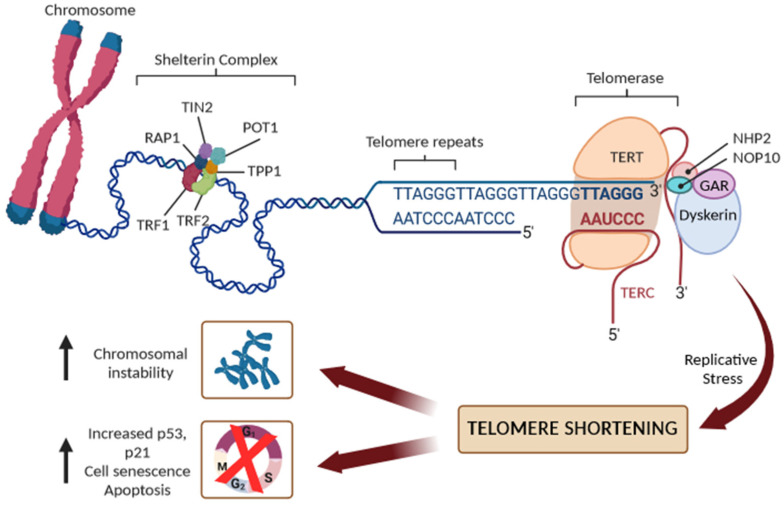
Schematic representation of telomere structure with the shelterin complex (TRF1, TRF2, TIN2, POT1, TPP1, and RAP1 proteins), and the main components of telomerase reverse transcriptase (TERT): its RNA component (TERC), the protein dyskerin, and other associated proteins (NHP2, NOP10, and GAR1). Replicative stress leads to progressive short dysfunctional telomeres, which cause end-to-end fusion of chromosomes and/or cell senescence or apoptosis.

Telomeres are nucleoprotein structures at chromosome ends that play a crucial role in protecting chromosomes from degradation, fusion, and erroneous recombination [6,7]. The telomeres comprise a variable number of double-stranded telomeric repeats, 5′-(TTAAGGG)n-3, in addition to a terminal region consisting of a single-stranded G-rich 3′ overhang, folding into higher-order structures to ensure the better protection of the chromosomal ends [6,7]. Indeed, the 3′-overhang would be recognized as a DNA double-strand break (DSB) and, thus, a target for DNA repair machinery, possibly leading to end-to-end telomere fusions. Consequently, telomeric DNA requires a protection mechanism, involving the formation of higher-order structures of telomeric DNA known as T-loops, sequestering the 3′ end of chromosomes and preventing its recognition by DNA damage sensors (Figure 1).

In humans, telomeres contain 2–15 kb of TTAGGG repeats, ending in 50–400 nucleotides of single-stranded overhang [6,7]. In the T-loop structure, telomeric repeats are bound by a specialized set of telomere-binding proteins, known as the “shelterin” complex, that are essential for telomere maintenance, chromosome integrity, and stability [8]. In human cells, the shelterin complex comprises six proteins: the TRF1 and TRF2 (telomeric repeat binding factor 1 and 2), the TIN2 (TRF1-interacting protein 2), POT1 (protection of telomeres protein 1), TPP1 (TIN2 and POT1 interacting protein), and the RAP1 (repressor/activator protein 1). The shelterin proteins form a complex with double-strand and single-strand DNA repeats at the telomere, and each shelterin component has a specific function in telomere protection. Overall, the shelterin complex, however, protects the telomeres from degradation, regulates the activity of telomerase, and prevents the activation of the DNA damage response, ensuring that senescent signaling pathways or apoptosis are not triggered [7,8]. To avoid the attrition of telomeres, germ cells, stem cells and some somatic cells produce telomerase reverse transcriptase (commonly referred to as telomerase) to maintain telomere length [6,7,8]. Human telomerase consists of a catalytic subunit, telomerase reverse transcriptase (TERT), which uses the telomerase RNA component (TERC) carrying a sequence complementary to the telomeric repeats to synthesize new telomeric DNA to the overhang. Due to the inability to maintain the length of the 3′ overhang, telomeres shorten by 30–150 base pairs, however, with each cell division [6,7,8]. Additionally, both endogenous and environmental risk factors may accelerate detrimental telomere shortening [4]. When a critical telomere length is reached, shelterin proteins cannot bind sufficiently, leading to reduced T-loop stability and protective function. Telomere-free ends emerge and they are recognized as DSBs, which activate the DNA damage repair system and cell-cycle inhibitors, including p16Ink4a, p21Cip1, and p53, leading to the activation of replicative senescence, which is followed by apoptosis. In the absence of cell-cycle checkpoint pathways, uncapped telomeres may cause the end-to-end fusion of chromosomes and multiple chromosome aberrations, causing an excess of genomic instability and a massive cell death (Figure 1).

## 3. Telomere Length and Atherosclerosis

It is well recognized that atherosclerosis is a chronic progressive inflammatory disease that results in a narrowed arterial lumen caused by arterial plaque accumulation, with various risk factors that contribute to its development [9]. When a plaque develops an unstable phenotype, it is prone to rupture, which can lead to major adverse cardiovascular events (myocardial infarction, stroke, and sudden death).

Despite the significant advances that have been made in understanding the risk factors and biological mechanisms of atherosclerosis, significant residual risk remains, and the rates of mortality and disability remain a severe threat to health in developed countries [10]. The occurrence of cardiovascular complications is unpredictable, and it is critically important to identify the cellular and molecular changes involved in the formation and destabilization of atherosclerotic plaques [11]. Additionally, it is fundamental to identify novel biomarkers for improving the stratification of patients at high risk of acute cardiovascular and cerebrovascular events [11].

At the present time, there is increasing evidence showing that premature or accelerated vascular senescence driven by telomere attrition and dysfunction contributes to atherosclerosis [4,12,13].

The attrition of telomeres has been reported in both endothelial cells (ECs) and vascular smooth muscle cells (VSMCs) with senescence-associated phenotypes, including senescence-associated β-galactosidase activity (SABG), in human and mouse models [4,12,13]. In human aortic endothelial cells, telomere dysfunction led to increased levels of intercellular adhesion molecule (ICAM)-1 expression and decreased endothelial nitric oxide synthase (eNOS) activity [4,12,13]. In a VSMC from human plaque, multiple markers of senescence and SABG were found to be associated with oxidative DNA damage and marked telomere shortening. Importantly, the senescence phenotype of vascular cells was significantly reversed by inducing the induction of telomerase activity [4,12,13].

From a clinical point of view, many of the studies included in meta-analyses have investigated the relationship between the leukocyte telomere length (LTL) and an increased risk of coronary artery disease (CAD), defined as non-fatal myocardial infarction, coronary heart disease death, or coronary revascularization, independent of conventional vascular risk factors [14,15,16]. Moreover, a significant association was found between shortened LTL and myocardial infarction and stroke, suggesting that LTL attrition may be a potential marker of plaque rupture and acute ischemic events [15]. Recently, large genome-wide association studies (GWAS) have identified genetic variants associated with LTL that have been used as instrumental variables in Mendelian Randomization (MR)-based approaches to confirm a causal relationship between a shorter TL and increased CAD risk [17,18]. Moreover, our group has shown, in patients with coronary artery disease, that a short LTL as well as an increased level of mtDNA alterations are predictors of adverse cardiovascular events and all-cause mortality [19,20]. Furthermore, a very large study, among a nationally representative US population (n = 7252), showed a moderate correlation between LTL and known cardiovascular risk factors, even after accounting for age, social and demographic factors, health behaviors, and white blood cell types [21]. Therefore, a short telomere length may be accelerated by cardiovascular risk factors and reflect the overall burden of inflammatory and oxidative stress involved in atherogenesis. Additionally, telomere erosion in endothelial progenitor cells (EPCs) originating from the hematopoietic stem cells (HSCs) pool may accelerate the rate of senescence in coronary endothelial cells [22], leading to less effective endothelial repair and the subsequent destabilization of atherosclerotic lesions, triggering the development of CAD and the occurrence of cardiovascular events.

**Figure 2:**
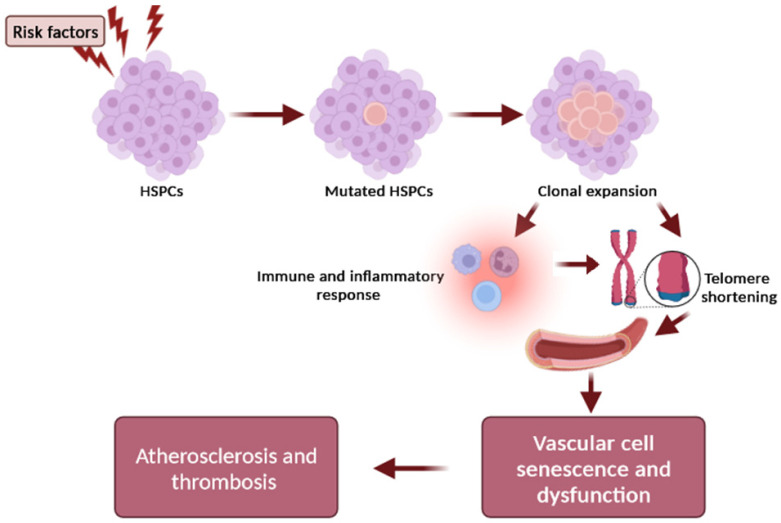
Schematic representation of the relationship between telomere and CHIP in atherogenesis. Risk factors (e.g., age, smoking, environmental exposures) influence the occurrence of a mutation in a CHIP driver gene of hematopoietic stem cells (HSCs), resulting in the expansion of a mutated HSC clone and mutant circulating leukocytes. The clonal expansion can promote inflammation and intensify telomere shortening in arteries, which further accelerates the adverse effects of telomere dysfunction on the vascular cell senescence and dysfunction, contributing to plaque formation and atherothrombotic risk.

Another pathophysiological mechanism that explains the causal relationship between telomere shortening and atherosclerosis development focuses on the clonal hematopoiesis of indeterminate potential (CHIP), which represents a new and independent risk factor for coronary artery disease, thrombosis, and heart failure [23,24]. CHIP is a common phenomenon that is strongly associated with aging and present in at least 4% of blood cells. It is a clonal expansion of blood stem cells based on somatic mutations (with a variant allele frequency ≥2%) in leukemia-associated genes, occurring in healthy people in the absence of a malignancy or cytopenia [23,24]. Altered inflammatory signaling from expanded leukocyte clones that carry somatic mutations may represent the major functional mechanism of plaque development and instability [24]. However, the causal relationship and underlying molecular alterations still require further research so as to define how CVD and clonal hematopoiesis are linked, as well as the potential clinical and therapeutic implications. Genetic association studies have shown that the most robust susceptibility locus for CHIP includes the TERT locus, which is also the lead locus influencing the LTL [25]. The association with the TERT locus supports a significant relationship between telomere biology and CHIP. Importantly, the prevalence of clonal hematopoiesis of indeterminate potential (CHIP) is higher in individuals with short LTL [25], suggesting that the dynamics of telomere length may be a critical mechanism in the relationship between CHIP and atherosclerosis [25]. Interestingly, a bidirectional Mendelian randomization study showed that a longer LTL promotes CHIP acquisition, whereas CHIP in turn shortens the LTL in affected cells, supporting the hypothesis that CHIP-associated CAD risk may be partly mediated by resultant LTL shortening [26]. The overview of telomere length, CHIP, and associated atherosclerosis is depicted in Figure 2.

However, the interplay between telomere biology and clonal hematopoiesis in atherosclerosis remains still largely undefined and underexplored, and it is critical that research better elucidates the clinical implications.

Nevertheless, available data support the notion that dysfunctional telomeres have a causal risk factor for vascular aging and atherogenesis. Therefore, the study of telomere biology is fundamental for both the prevention of and the development of treatment for atherosclerosis and other age-related diseases.

## 4. TERRA Transcription

**Figure 3:**
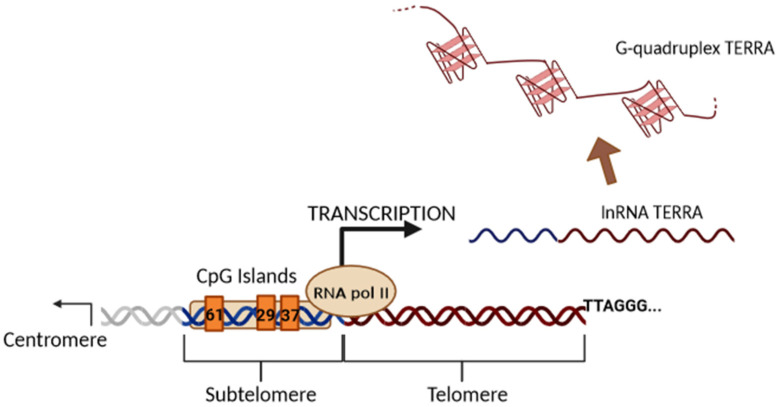
Illustration of the biogenesis of TERRA generated via transcription. The black arrow indicates the direction of transcription by RNAPII from the subtelomeric sequences (61-29-37 repeats TERRA promoter) into the telomeric tract. TERRA transcripts are indicated as soluble or stabilized G-quadruplex structures.

Until 2007, telomeres were assumed to be transcriptionally silent due to their heterochromatic state and low gene density. Subsequently, it was discovered that RNA polymerase II initiates transcription in subtelomeric regions to produce the evolutionarily conserved long noncoding TERRA in mouse and human cells [5,27], changing the previous view and stimulating new research in the telomere field [28,29,30]. Later, it was shown that TERRA is expressed in many other species, demonstrating its conservative nature [28,29,30]. TERRA transcription begins from the subtelomeric region in the 5′ to 3′ direction (from the centromere to telomere) and proceeds to the end of chromosomes within the telomeric repeats (Figure 3).

**Figure 4:**
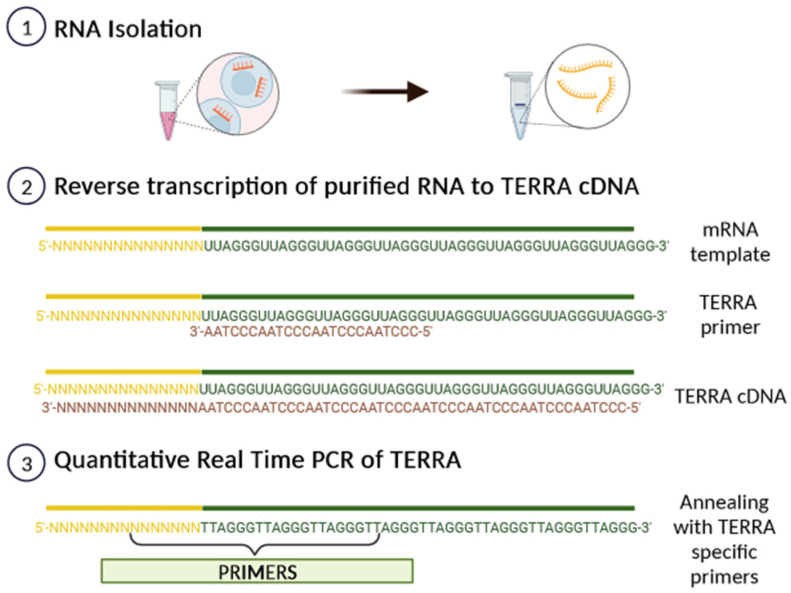
A scheme of TERRA analysis by qRT-PCR. After RNA isolation, TERRA-specific primers can generate a pool of TERRA cDNA molecules with diverse subtelomeric sequences. By using PCR-specific primer pairs, it is possible to amplify the subtelomeric sequence from each chromosome from the pool of transcribed TERRA cDNA.

Subtelomeric CpG islands, consisting of three different repetitive DNA sequences known as “61-29-37 bp repeats”, are thought to act as TERRA promoters and binding sites for the chromatin organizing factor CTCF (CCCTC-binding factor), which acts as a key regulator of TERRA expression [31]. TERRA transcripts are heterogeneous in length, ranging anywhere from 100 bases up to 9 kilobases in length for human cells, consist of both subtelomeric-derived sequences and a G-rich telomeric repeat tract with an average length of 200 bases, and they may be stabilized by a G-quadruplex forming at the displaced G-rich DNA strand. TERRA is mostly detected in nuclear cellular fractions, where they colocalize with telomere in the form of RNA-DNA hybrid R-loop [28,29,30]. TERRA 5′ ends are capped by 7-methyl-guanosine cap structures and rarely (~10%) polyadenylated at their 3′. This polyadenylation influences TERRA stability and localization, supporting the notion that different fractions may have different functions [28,29,30]. Non-polyadenylated TERRA mainly localizes to telomeres, while polyadenylated TERRA is found in the nucleoplasm, exhibiting a much longer half-life than the non-polyadenylated form with a half-life of 8 to less than 3 h, respectively [28,29,30]. Moreover, it has been also shown that TERRA is regulated in a cell-cycle-dependent manner in human cells. TERRA levels are higher in G1 and progressively decline upon entry into the S phase of the cell cycle, reaching the lowest levels in the late S/G2 phase, a time that roughly corresponds to telomere replication [28]. Additionally, Feretzak and colleagues demonstrated that TERRA is expressed from different chromosome ends and in a variety of normal and several cancerous human cell lines by using real-time RT-PCR quantification [32,33]. Both the subtelomeric-derived sequence and telomere-derived TERRA can be analyzed via RT-qPCR using specific primers, allowing one to distinguish the chromosome of origin of a TERRA transcript, although some chromosomes have highly similar subtelomeric sequences (Figure 4).

## 5. Biological Functions of TERRA

Recent observations support the notion that TERRA sustains multiple functions at telomeres, as has been extensively reviewed [29,30,34,35,36,37]. However, the complex biological functions of TERRA and the specific mechanisms by which TERRA contributes to telomere biology remain largely elusive. At present, TERRA is generally believed to play a role in the protection of telomeres, the regulation of telomere length and telomerase activity, as well as in the formation of heterochromatin at the ends of chromosomes [29,30,34,35,36,37]. Regarding TERRA’s interaction with telomerase, it can act as a negative regulator of telomerase activity in cells with long telomeres, while functioning as a positive regulator of telomerase activity at short telomeres [36]. In the presence of long telomeres, TERRA may act as a negative regulator of telomerase activity by acting through the base pairing of the tandem repeats found throughout TERRA’s 3′ end to the complementary RNA template region of the TERC region, and blocking telomerase binding to telomeric ssDNA [36]. Additionally, TERRA may also interact with the subunit hTERT as an allosteric inhibitor of telomerase function, abolishing the catalytic activity of adding further telomeric DNA repeats [36]. On the other hand, short telomeres may induce TERRA expression, which might represent a signal to recruit telomerase and perform subsequent telomere elongation [29,36].

**Figure 5:**
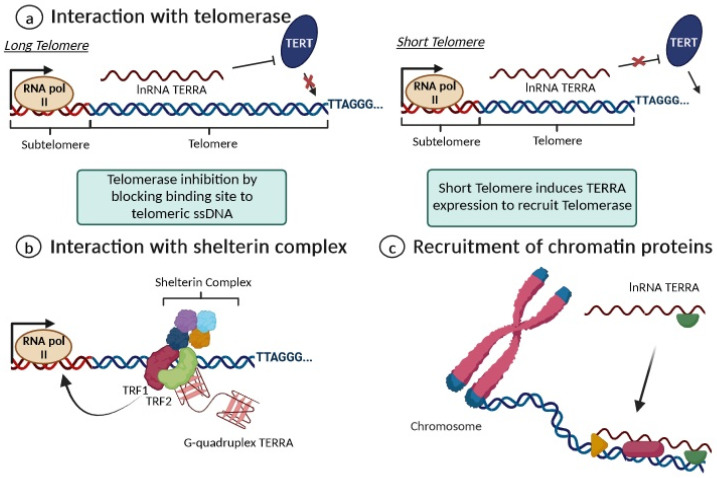
Overview of the main functions of TERRA. (**a**) For long telomeres (**left**), TERRA can bind to telomerase, repressing its activity; when a telomere is critically short (**right**), the repression of TERRA expression is removed. (**b**) TERRA interacts with shelterin proteins by enhancing chromosome-end protection with secondary protective structures, including G-quadruplexes; (**c**) TERRA contributes to maintaining heterochromatin formation via the recruitment of chromatin proteins.

Additionally, TERRA interacts with the shelterin complex, and its transcription is regulated by TRF1 and TRF2 [38,39,40]. Indeed, the G-quadruplex structure of TERRA is an important recognition element for the TRF2 shelterin subunit and physically interacts with it to bind to telomeric DNA and also with TRF1 to preserve the telomere’s structural stability [38,39,40]. By interacting with RNA polymerase II, TRF1 seems to positively regulate TERRA expression levels, while telomere dysfunction induced by the depletion of TRF2 leads to increased TERRA levels at all transcribed subtelomeres [38,39,40]. Moreover, long non-coding RNAs are known to mediate epigenetic changes on chromatin by functioning as recruiters for chromatin-modifying enzymes to genomic loci [29]. Accordingly, TERRA is thought to play a similar role, recruiting both heterochromatic proteins (H3K9me3 and HP1 proteins) and the associated chromatin-remodeling complexes to telomeres, such as NoRC (nucleolar remodeling complex), MORF4L2 (a component of the NuA histone acetyltransferase complex), and ARID1A (a component of the SWI/SNF nucleosome remodeling complex); this contributes to establishing or maintaining heterochromatin formation [29,39]. Figure 5 summarizes the key biological functions proposed for TERRA as a key regulator of telomere maintenance and heterochromatin formation in telomeres.

## 6. Dysregulation of TERRA in Human Studies

The study of TERRA dysregulation may be particularly relevant to the development and progression of several diseases in which telomere dysfunction is present. The first evidence of TERRA expression in humans comes from patients with facial anomalies syndrome (ICF), a rare autosomal recessive immune disorder caused by mutations in the DNA methyltransferase 3 [41]. Primary fibroblasts from ICF patients have shown hypo-methylated TERRA subtelomeric promoters and increased levels of TERRA, suggesting that this dysregulated expression may explain the abnormally short telomeres and cell senescence in ICF patients [41]. Several studies have shown the dysregulation, mainly the downregulation, of TERRA in various human cancer tissues compared with normal tissues [42,43,44,45,46,47,48]. Table 1 reports a summary of studies describing the expression profile of TERRA in different human studies.

Recently, a cell-free form of TERRA (cfTERRA) was also found to be a component of extracellular microvesicular exosomes in cancer cell culture and human blood plasma, as damage signals mediating the crosstalk between telomere dysfunction and inflammatory cytokines can lead to the activation of inflammation in the tissue microenvironment [49]. In addition, TERRA expression levels significantly increased in the peripheral blood mononuclear cells of patients with idiopathic pulmonary fibrosis (IPF) and were inversely correlated with force vital capacity (FVC%). Remarkably, this study also used a cell model to demonstrate that TERRA might contribute to IPF pathogenesis, and that RNA interference on TERRA can improve telomeric and mitochondrial functions [50]. In healthy young volunteers, cycling endurance exercise, which is associated with AMPK activation, induced an up-regulation of TERRA in skeletal muscle biopsies obtained from 10 volunteers, supporting the notion that exercise may positively affect telomeres by promoting telomerase recruitment in telomeres and by preventing the aging of postmitotic tissues and stem cells [51]. Finally, a decreased level of TERRA expression was observed in sarcopenic subjects when compared to non-sarcopenic controls. However, an intervention combining exercise training and nutrition supplementation increased TERRA expression in participants with sarcopenia [52]. Altogether, these findings support the intriguing possibility that TERRA could be a great potential biomarker and therapeutic target in the clinical management of several diseases.

## 7. TERRA Expression and Vascular Biology

Although it is well documented that telomere dysfunction is involved in vascular and cardiac cell senescence [1,2,3,4], the role of TERRA in vascular biology is surprisingly unknown. To date, no study has investigated the association between the TERRA expression profile and cardiovascular diseases, especially for the clinical conditions in which there is strong evidence of the relationships between telomere shortening and increased risk, such as coronary atherosclerosis, myocardial infarction, ischemic heart disease, and stroke [14,15,16,17,18,19,20]. Understanding TERRA transcription dynamics and its involvement in telomere biology may have a clear potential impact on clinical practice and the potential development of effective targeted vascular aging therapies. Indeed, therapeutic approaches to modulate the telomere system are considered as promising alternative treatments for cardiovascular pathologies [53,54]. The induction of telomerase activity, either after the reactivation of endogenous TERT expression, is considered a prime target in the development of potent therapeutics against vascular aging and cardiovascular disease [55]. Enhanced TERT activity can induce beneficial and protective effects through the canonical pathway against telomere shortening and the excessive senescence of vascular cells. Activating the non-canonical functions of TERT may protect mitochondrial function from antagonizing oxidative stress, DNA damage, and apoptosis [55]. Experimental evidence supporting this notion shows the therapeutic potential of enhancing the telomerase activity for cardiovascular disease [56,57]. For instance, telomerase gene transfer therapy improved ventricular function and prevented heart failure after acute myocardial infarction in mouse models [56]. Additionally, the use of the telomerase activator TA-65, a natural plant compound, has been reported to up-regulate telomerase activity and elongate telomeres without increasing cancer incidence [57]. However, despite these interesting results in preclinical models of aging and disease, the overexpression of TERT may have anti-apoptosis and cell immortalization effects, potentially increasing the risk of cancer [58]. The development of safe strategies in which telomerase is induced temporarily and selectively in aged cells without promoting off-target effects is essential to clarify its potential as a therapeutic target. One attractive application is the use of RNA-centered approaches specifically directed to TERRA and TERC, including RNA interference, antisense oligonucleotides and small molecules [59]. For instance, several studies have shown that drugs targeting TERRA molecules could be promising therapeutic strategies against cancer. For instance, TERRA expression in human cancer cells could also be regulated by demethylating agents and histone deacetylase inhibitors, such as 5-azacytidine and trichostatin [60,61]. Moreover, stabilizing the G4 structures of TERRA by using small-molecule ligands (e.g., BRACO-19, Telomestatin) may be one of the most promising approaches to inhibiting telomerase and hence inhibiting the proliferation of cancer cells and tumor growth [60,61]. Indeed, a recent in vitro and in vivo studies have shown that targeting TERRA using hit 17, a small molecule that can bind and stabilize the G4 conformation, could represent a promising strategy for a novel therapeutic approach to multiple myeloma [62]. Additionally, the use of a telomeric antisense oligonucleotide has been recently shown to selectively inhibit the DNA damage response in telomeres in both cells and mice, reducing the markers of cellular senescence apoptosis and the expression of SASP cytokines, as well as improving health and lifespan in an animal model [63,64]. Thus, studying the dynamics of TERRA and the antisense approach to targeting specific targets in human vascular cells and in vivo models may unveil unexpected functions for delaying vascular aging and also provide cardioprotection.

## 8. Conclusions

In summary, it is well documented that TERRA is a key regulator in telomere maintenance, regulating telomerase activity and heterochromatin formation. However, the multiple biological functions of TERRA and its mechanisms of action are still to be fully discovered. In particular, there is no knowledge of the molecular mechanisms by which TERRA may regulate telomere homeostasis in vascular and cardiomyocyte cells. Further experimental and clinical studies are of great interest in cardiovascular research, as TERRA regulation is altered in several human diseases. More work is also needed to better understand the connection between CHIP and telomere dysfunction, and how they are linked to differential expression changes in TERRA in vascular aging and atherosclerosis. A comprehensive understanding of the TERRA transcripts may provide valuable insights into telomere-associated vascular aging, offering great potential for new therapeutic approaches.

## Figures and Tables

**Table 1:** Summary of human studies with TERRA expression profile and dysregulation.

Reference	Human Model	Study Design	Biological Sample	Method	Main Findings
Sampl S et al., 2012 [42]	Astrocytoma	Cancer tissue	Tissue	Real-Time PCR	Down-regulation
Vitelli V et al., 2018 [43]	Head and neck squamous cell carcinoma	Healthy tissue/Cancer tissue	Tissue	Real-Time PCR	Down-regulation
Adishesh M et al. 2020 [44]	Endometrial cancer	Healthy tissue/Cancer tissue	Endometrial biopsy	Real-Time PCR	Down-regulation
Bae SU et al., 2019 [45]	Colorectal cancer	Cancer tissue	Tissue	Real-Time PCR	Up-regulation
Storti CB et al., 2020 [46]	Non-small cell lung cancer	Healthy tissue/Cancer tissue	Tissue	Real-Time PCR	Up-regulation
Cao H et al., 2020 [47]	Hepatocellular carcinoma	Healthy tissue/Cancer tissue	Tissue	FISH	Down-regulation
Manganelli M et al., 2022 [48]	Hepatocellular carcinoma	Healthy tissue/Cancer tissue Case/Control	Tissue Plasma	Real-Time PCR	Down-regulation in tissue Up-regulation in plasma
Wang Z et al., 2015 [49]	Cancer	Case/Control	Plasma	RNA Seq	Up-regulation
Gao Y et al., 2017 [50]	Idiopathic pulmonary fibrosis	Case/Control	Blood	Real-Time PCR	Up-regulation
Diman A et al., 2016 [51]	Healthy runners	Pre/post exercise tissue	Muscle biopsy	FISH	Up-regulation
Chang KV et al., 2020 [52]	Sarcopenia	Case/Control	Leukocytes	Real-Time PCR	Down-regulation
